# Development and application of a qPCR assay for *Lawsonia intracellularis* in Tibetan pigs

**DOI:** 10.3389/fcimb.2025.1711578

**Published:** 2025-12-03

**Authors:** Chendong Xiao, Yanqiong Wang, Tingyu Chen, Lihong Xue, Hongyan Zhao, Changyou Xia, Xiaogang Zhao

**Affiliations:** 1 Center for Animal Disease Control and Prevention in Xiangcheng, Xiangcheng, Sichuan, China; 2 State Key Laboratory for Animal Disease Control and Prevention, Harbin Veterinary Research Institute, Chinese Academy of Agricultural Sciences, Harbin, China; 3 Agriculture, Animal Husbandry, Rural and Science and Technology Bureau of Xiangcheng, Xiangcheng, Sichuan, China

**Keywords:** Tibetan pig, *Lawsonia endocellularis*, *aspA*, quantitative PCR, epidemiology

## Abstract

This study developed a rapid, sensitive, and specific TaqMan MGB based qPCR assay for detecting *Lawsonia intracellularis* in Tibetan pigs by targeting the *aspA* gene. The method showed no cross-reactivity with other common porcine enteric pathogens, and exhibited high sensitivity (detection limit: 7.056 copies/μL) and excellent repeatability (CV < 2%). A standard curve with good linearity (R² = 0.998) and 110.95% amplification efficiency was established. The assay was applied to 237 fecal samples collected from Tibetan pigs across eight townships in Xiangcheng County, Sichuan Province, in parallel with a national standard method. Results indicated a 19% overall positivity rate (45/237), with regional rates ranging from 11.11% to 26.67%. The established qPCR provides a reliable tool for field surveillance, and findings underscore the need to integrate *L. intracellularis* monitoring into local prevention systems to support healthy Tibetan pig production.

## 1 Introduction

Porcine proliferative enteropathy (PPE), also known as porcine ileitis, is an important intestinal infectious disease caused by *Lawsonia intracellularis* (*L. intracellularis*) *(*Campillo et al., 2021). The clinical manifestations can be divided into three forms: acute, chronic, and subclinical. The acute form, primarily seen in pigs aged 4–12 months, is characterized by hemorrhagic proliferative enteritis with clinical signs including bloody diarrhea, tarry feces, and even sudden death (Obradovic and Wilson, 2020). The chronic form, often occurring in growing pigs aged 6–20 weeks and referred to as porcine intestinal adenomatosis, presents with intermittent diarrhea, reduced appetite, growth retardation, and decreased herd uniformity (Obradovic and Wilson, 2020). The subclinical form, which is the most common, typically shows no obvious symptoms but still leads to reduced growth performance and economic losses (Campillo et al., 2021). It is estimated that feed cost increases by $2–20 per infected pig, posing a substantial economic burden on the global pig industry (Mcorist et al., 1997). For instance, annual losses in the United States and the United Kingdom reach approximately $20 million and £2–4 million, respectively (Mcorist et al., 1997).

*L. intracellularis* is a Gram-negative, microaerophilic, obligate intracellular bacterium (Xiao et al., 2022). The bacilli are curved or straight rods measuring approximately 1.25–1.75 μm in length and 0.25–0.43 μm in width, and do not form spores. This bacterium cannot be cultured on conventional media and requires eukaryotic cell lines (e.g., IEC-18, IPEC-J2) for *in vitro* propagation (Xie et al., 2025). Its isolation and maintenance are technically challenging, leading to relatively underdeveloped detection methods and somewhat limiting in-depth research. Serological and molecular surveys indicate that *L. intracellularis* infection is highly prevalent in pig farms worldwide. As the world’s largest producer of pigs and pork, China also shows a high infection rate, with some reports even exceeding 90% (Wang et al., 2024). However, epidemiological data in specific local pig breeds—particularly Tibetan pigs—remain scarce.

Xiangcheng County in Ganzi Prefecture, Sichuan Province, is located in a high-altitude mountainous region. Its local breed, the Xiangcheng Tibetan pig, is well-adapted to high-altitude environments, resistant to rough feeding, and known for excellent meat quality, making it an important genetic resource and a distinctive local industry (Yang et al., 2024). Yet, the health status of this herd is largely unknown, and no information is available on *L. intracellularis* infection. This complete absence of data, coupled with the known economic impact of PPE, underscores the critical necessity for a reliable detection method to facilitate timely and accurate diagnosis and effective intervention. Thus, we established a qPCR assay for *L. intracellularis* and applied it to fecal samples from Tibetan pig farms across eight townships in Xiangcheng County to investigate the local prevalence. Our results fill a key knowledge gap and offer a practical tool for early detection and control of *L. intracellularis* in this important pig breed, which is crucial for mitigating economic losses and ensuring sustainable production.

## 2 Materials and methods

### 2.1 Nucleic acids and clinical samples

Nucleic acids of *L. intracellularis*, Enteropathogenic *Escherichia coli* (EPEC), *Salmonella Choleraesuis*, *Pasteurella multocida*, *Clostridium perfringens*, Porcine epidemic diarrhea virus (PEDV), Transmissible gastroenteritis virus (TGEV), Porcine group A rotavirus (PoRVA), and Porcine delta coronavirus (PDCoV) were preserved in our laboratory. A total of 237 fecal samples from Tibetan pigs were collected from eight townships in Xiangcheng County, Ganzi Tibetan Autonomous Prefecture, Sichuan Province, including Ranwu Township (n = 30), Shuiwa Township (n = 22), Reda Town (n = 18), Zhengdou Township (n = 20), Baiyi Township (n = 30), Shagong Standard Farm (n = 50), Xiangbala Town (n = 30), and Qingde Town (n = 37). A total of 237 fecal samples of healthy Tibetan pigs were collected from some farms in Xiangcheng County, Garze Prefecture. It is imperative to underscore that no additional harm or intervention was imposed on the animals involved in this study. The Institutional Review Board of the Harbin Veterinary Research Institute has determined that this study is exempt from the requirement for ethical review or approval.

### 2.2 Primer design

Based on the *aspA* gene of *L. intracellularis* (GenBank ID: CP107054.1), primers and a probe for qPCR, as well as primers for constructing the recombinant plasmid standard, were designed using Primer Express 3.0.1 and Primer Premier 5 software (**Table 1**). All primers and the probe were synthesized by Sangon Biotech (Shanghai) Co., Ltd. and working concentrations were diluted to 10 μM. The 1118 bp fragment used to construct the recombinant plasmid standard fully contains the 159 bp qPCR target region.

**Table 1:** Primer and probe sequence.

Pathogen	Gene	sequence(5′-3′)	Product size (bp)
*L. intracellularis*	*aspA*	LI-F: TGGTCCTCGCTGTGGATTG LI-R: GCCTCTGCTGCCATTGTGA Probe:FAM- CCCGGTTATTCCAGAAGT-MGB	159
		Z-F: CTTATTATGGCTGTCAAACACTCC Z-R: AATACCGTTGAAACATTTATCTGC	1118

### 2.3 Construction of recombinant plasmid standard

The recombinant plasmid standard was constructed by amplifying the target gene from *L. intracellularis* using primers Z-F/Z-R. The PCR product was purified, cloned into pMD19-T vector, and transformed into *E. coli* DH5α. After sequencing verification, positive clones were cultured and the plasmid was extracted. Determination of plasmid concentration using Nanodrop. The copy number was determined spectrophotometrically and calculated using the formula:Copies/µL = (6.02 × 10²³) × (concentration in ng/µL × 10⁻^9^)/(plasmid length in bp × 660) (Tian et al., 2025).

### 2.4 Optimization of qPCR conditions

The qPCR assay was optimized using a single-variable approach for primer concentration, probe concentration, annealing temperature, and cycle number. The reaction mixture consisted of: 10 μL of 2× TaqMan Fast qPCR Master Mix, 0.1–0.6 μM each of LI-F and LI-R primers, 0.1–0.6 μM probe, 2 μL of template DNA, and nuclease-free water added to a final volume of 20 μL. The thermal cycling conditions were as follows: pre-denaturation at 95°C for 30 s, followed by 35–50 cycles of denaturation at 95°C for 10 s and combined annealing/extension at 58–63°C for 30 s.

### 2.5 Assay validation

The specificity of the qPCR assay was confirmed using genomic DNA or cDNA from common swine enteric pathogens, including EPEC, *Salmonella Choleraesuis*, *Pasteurella multocida*, *Clostridium perfringens*, PEDV, TGEV, PoRVA, and PDCoV. *L. intracellularis* nucleic acid and nuclease-free water were used as positive and negative controls, respectively. For sensitivity evaluation, a standard curve was generated using 10-fold serial dilutions of the recombinant plasmid standard (from 0.5×10^7^ to 0.5 copies/μL), with each dilution tested in triplicate. The limit of detection (LOD) was determined by probit regression analysis (IBM SPSS Statistics 25) at 95% confidence level using 40 replicates of low-copy samples (ranging from 100 to 3.125 copies/μL) (Tian et al., 2025). Repeatability was assessed through intra- and inter-assay tests using standards at 10^7^, 10^5^, and 10³ copies/μL, with results expressed as mean Ct and coefficient of variation (CV).

### 2.6 Clinical sample testing

Genomic DNA was extracted from 237 Tibetan pig fecal samples using an automated nucleic acid extraction system. Both the newly developed qPCR assay and the method specified in the Chinese industry standard SN/T 3488-2013 (using primers and probe for *Lawsonia intracellularis* detection) were applied in parallel to evaluate the accuracy of the established assay (Quarantine protocol for real-time PCR of Lawsona intracellularis). Additionally, the prevalence of *L. intracellularis* across different townships in Xiangcheng County was investigated.

## 3 Results and analysis

### 3.1 Optimization of qPCR conditions

To improve amplification efficiency, key parameters including primer/probe concentration, annealing temperature, and cycle number were optimized using a single-variable approach. Optimal results were achieved with 0.3 μM primers, 0.2 μM probe, and an annealing temperature of 59°C, yielding low Ct values and high efficiency. Cycle number optimization revealed that 35 cycles resulted in insufficient amplification, whereas more than 40 cycles led to non-specific amplification and an elevated background. Therefore, 40 cycles were selected for subsequent experiments.

### 3.2 Specificity validation

**Figure 1:**
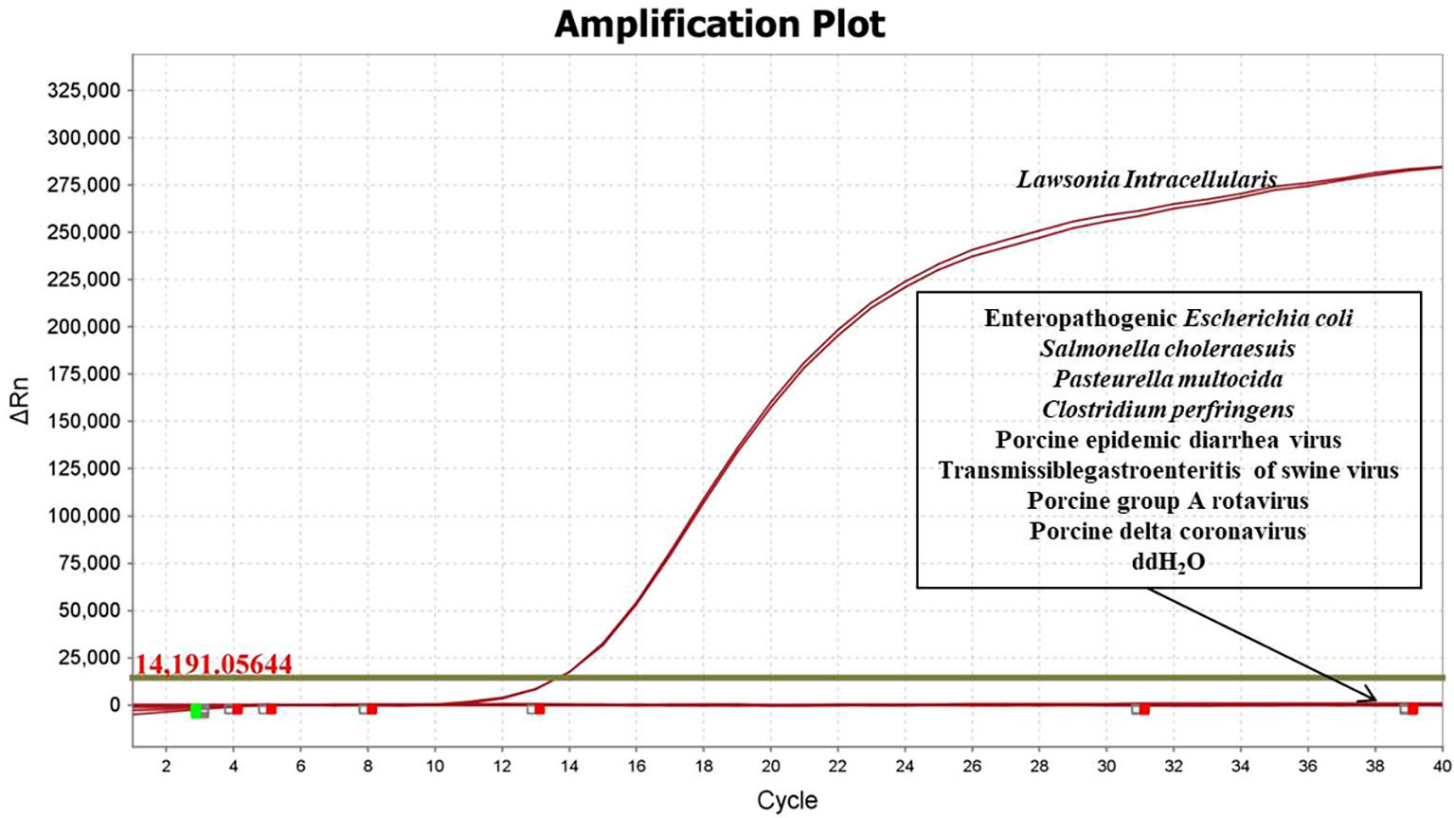
Specific verification results of fluorescence qPCR method for *L. intracellularis*.

The specificity of the developed qPCR assay was evaluated using DNA/cDNA from EPEC, *Salmonella Choleraesuis*, *Pasteurella multocida*, *Clostridium perfringens*, PEDV, TGEV, PoRVA, and PDCoV. The results demonstrated that amplification occurred only in the presence of *L. intracellularis* nucleic acid, with no cross-reactivity observed with any of the other tested pathogens (**Figure 1**), confirming high specificity of the assay.

### 3.3 Standard curve

**Figure 2:**
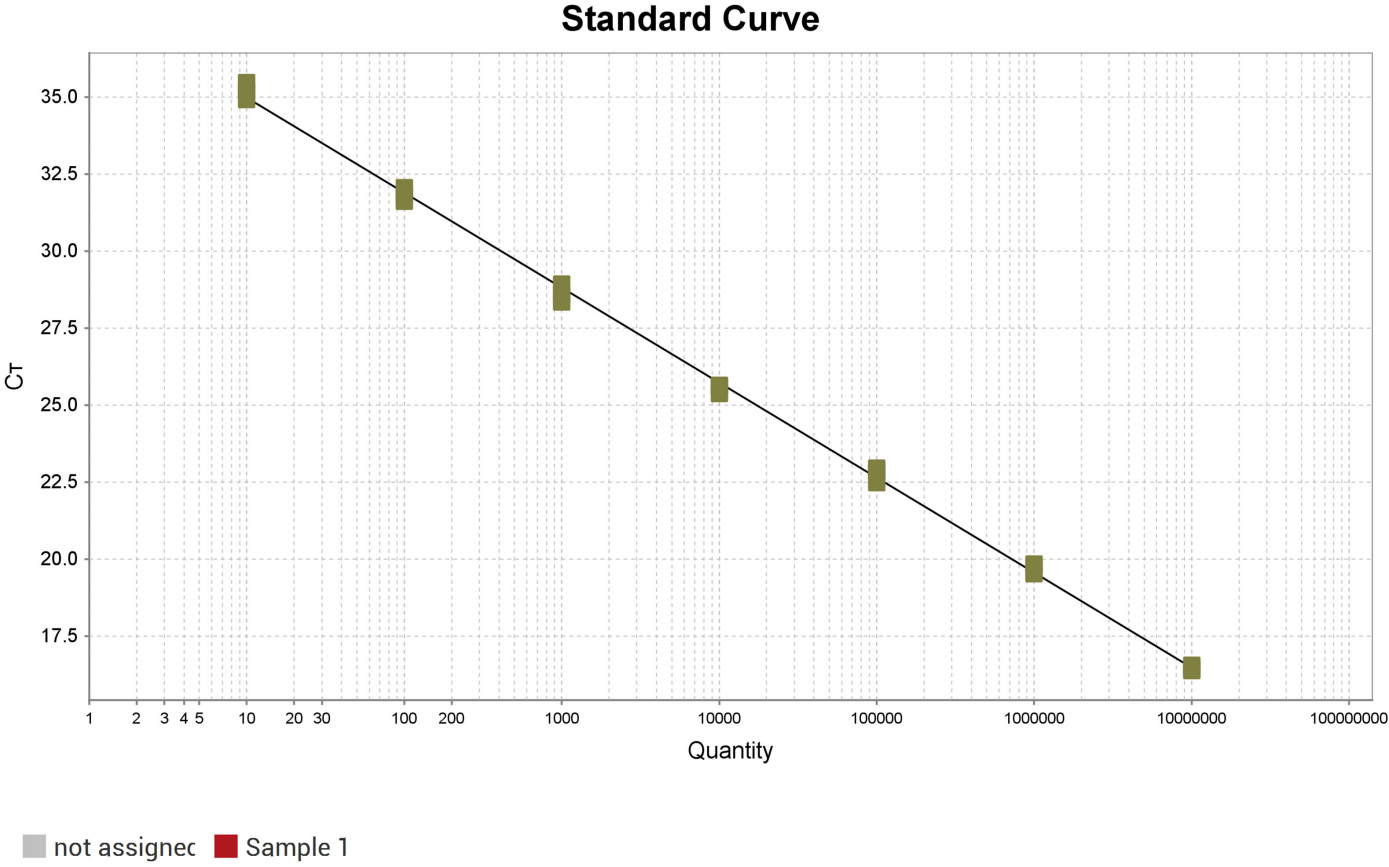
Standard curve of fluorescence qPCR method for *L. intracellularis*.

A standard curve was generated using 10-fold serial dilutions of the recombinant plasmid, ranging from 0.5×10^7^ to 0.5 copies/μL. The plasmid copy number (X-axis) and Ct values (Y-axis) were plotted using QuantStudio™ Design & Analysis Software v1.3.1. The assay demonstrated excellent linearity (R² = 0.998) and high amplification efficiency (110.95%), with a linear regression equation of Y = –3.085log(X) + 38.07 (**Figure 2**).

### 3.4 Sensitivity and limit of detection

**Figure 3:**
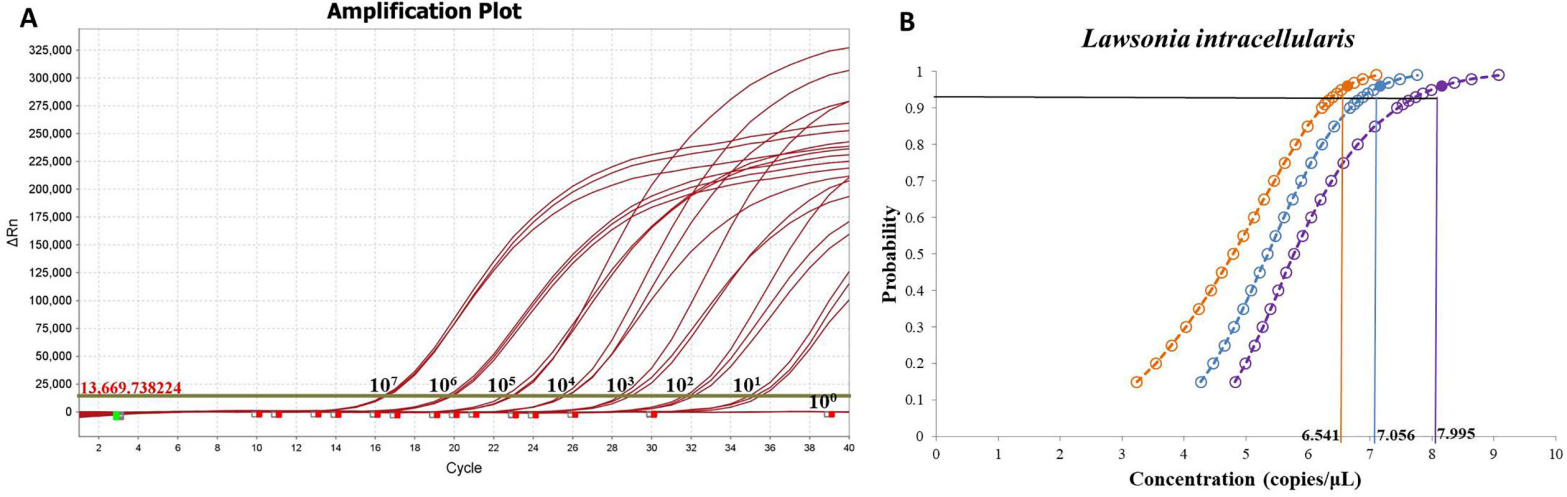
Sensitivity and limit of detection of fluorescence qPCR method for *L. intracellularis.***(A)** Detection of plasmid standards from 10^7^ to 10^0^ copies using fluorescence quantitative PCR method; **(B)** Probability regression analysis of fluorescence qPCR.

The sensitivity of the fluorescence qPCR assay was evaluated by testing serial dilutions of the recombinant plasmid standard. The results indicated that the method could reliably detect as low as 10 copies (**Figure 3A**). Determine the approximate range of sensitivity through gradient dilution detection. To determine the limit of detection (LOD), gradients ranging from 100 to 3.125 copies/μL were tested with 40 replicates each. Probit regression analysis (IBM SPSS Statistics 25) established the LOD at 7.056 copies/μL (95% CI: 6.541–7.995) (**Figure 3B**), with detailed average Ct values and detection rates shown in **Table 2**. The assay was considered valid when positive controls produced typical amplification curves and negative controls showed no amplification (Ct = 40 or undetermined). A sample was determined positive for *L. intracellularis* if the FAM channel exhibited a typical amplification curve with a Ct value ≤ 35.4. Samples with Ct values between 35.4 and 40 were considered suspected positives and were retested with a doubled template volume. A sample was confirmed positive if the Ct value in the retest was below the threshold; otherwise, it was deemed negative. Samples with no amplification in the FAM channel (Ct = 40 or undetermined) were classified as negative for *L. intracellularis*.

**Table 2:** shows the average Ct values and hit rates for the detection of continuously diluted plasmid standards.

Positive plasmid	Concentration (copy/μ L)	Sample quantity	Fluorescence qPCR method	
			Ct (average)	Hit rate (%)
pMD19-aspA	100	40	31.850	100
	50	40	32.904	100
	25	40	33.647	100
	12.5	40	34.728	100
	6.25	32	35.665	80
	3.125	0	Undetermined	0

### 3.5 Repeatability

To evaluate the reproducibility of the assay, intra- and inter-assay tests were performed using plasmid standards at concentrations of 10^7^, 10^5^, and 10³ copies/μL. The coefficients of variation (CV) for both intra- and inter-assay replicates were below 2% (**Table 3**), demonstrating high repeatability and stability of the established fluorescence qPCR method.

**Table 3:** Repeatability detection results of fluorescence qPCR method.

Positive plasmid	Concentration (copies/μL)	Batch testing		In batch testing	
		X ± SD	CV (%)	X ± SD	CV (%)
pMD19-aspA	10^7^	16.411 ± 0.032	0.19	16.816 ± 0.014	0.08
	10^5^	22.655 ± 0.164	0.72	22.439 ± 0.090	0.40
	10^3^	28.715± 0.334	1.16	28.622 ± 0.199	0.70

### 3.6 Sample testing results

**Figure 4:**
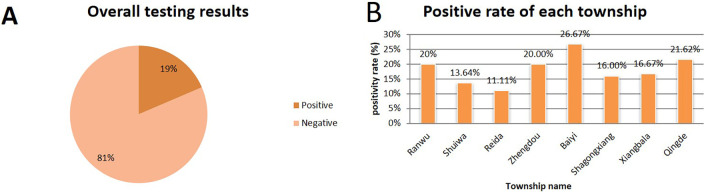
Test results of 237 Tibetan pig manure samples.

To evaluate the clinical applicability of the established method and investigate the prevalence of *L. intracellularis* in Tibetan pig farms across Xiangcheng County, Ganzi Prefecture, a total of 237 fecal samples were tested using both the newly developed qPCR assay and a previously published standard method. The results showed an overall positivity rate of 19% and a negativity rate of 81% (**Figure 4A**). The positivity rates in Ranwu Township, Shuiwa Township, Reda Town, Zhengdou Township, Baiyi Township, Shagong Standard Farm, Xiangbala Town, and Qingde Town were 20%, 13.64%, 11.11%, 20.00%, 26.67%, 16.00%, 16.67%, and 21.62%, respectively (**Figure 4B**). The detection results of these two methods have a 100% agreement rate.

## 4 Discussion

Porcine proliferative enteropathy (PPE), caused by *Lawsonia intracellularis*, is a globally significant infectious intestinal disease associated with substantial economic losses in the swine industry (Wang et al., 2024). This study developed a TaqMan-based qPCR assay targeting the *aspA* gene of *L. intracellularis* and conducted the first molecular epidemiological investigation of this pathogen in Tibetan pigs from Xiangcheng County, Ganzi Tibetan Autonomous Prefecture, Sichuan Province.

The established fluorescence qPCR method demonstrated excellent performance, including high specificity with no cross-reactivity with other common swine enteric pathogens, high sensitivity with a detection limit of 7.056 copies/μL (95% CI: 6.541–7.995), a wide linear range (10^7^ to 10¹ copies/μL) with good linear correlation (R² = 0.998), and high repeatability with both intra- and inter-assay coefficients of variation below 2%. Compared with the current industry standard method (SN/T 3488-2013), the developed assay showed improved sensitivity and stability while maintaining high consistency, making it suitable for application in basic laboratories.

Epidemiological investigation revealed an overall positivity rate of 19% (45/237) in Tibetan pigs from Xiangcheng County, indicating a certain degree of endemicity. Positivity rates varied among townships, ranging from 11.11% (Reda Town) to 26.67% (Baiyi Township). This rate is lower than many reports from commercial pig herds domestically and internationally. For example, Wang et al. reported a fecal positivity rate of 37.3% (95% CI: 34.1–40.5%) and a farm-level positivity rate of 93.6% (95% CI: 65.3–94.4%) using qPCR (Wang et al., 2024). Notably, the highest positivity rate (26.67%) was observed in Baiyi Township, where samples were collected from a local intensive farm. In contrast, the lower rates in other townships were associated with free-range or backyard farming practices. This striking disparity strongly suggests that the discrepancy in overall prevalence may reflect the unique characteristics of Tibetan pigs as a local breed, which are typically raised under low-density free-range or semi-intensive systems in high-altitude regions, resulting in reduced pathogen exposure and transmission pressure. The intensive farming conditions in Baiyi Township, characterized by higher animal density, likely facilitated fecal-oral transmission of L. intracellularis, aligning it more closely with the epidemiological patterns observed in commercial herds (Wang et al., 2024). Although the overall infection rate was not high, positive samples were detected in all surveyed townships, indicating that *L. intracellularis* is widely present in the local Tibetan pig farming environment. Subclinical infections are common in PPE and can lead to reduced feed efficiency and impaired growth performance even in the absence of obvious diarrheal symptoms (Wang et al., 2024; Hu et al., 2025). Therefore, although no large-scale outbreaks have been reported in Xiangcheng Tibetan pigs, the potential economic losses should not be overlooked, especially as farming practices shift toward standardization and intensification, which may increase infection pressure. Continuous monitoring and integration into routine disease prevention systems are necessary. This study has several limitations. The sample size was relatively small (n = 237) and collected during a single time period, which may not reflect seasonal dynamics or age-related differences. Additionally, the lack of bacterial isolation prevented genetic characterization of local strains.

Regarding the qPCR assay used in this study, the calculated amplification efficiency was 110.95%, which is slightly above the ideal range of 90-110%. This elevated efficiency is most likely attributable to minor inaccuracies during the serial dilution of the standard DNA for the standard curve. Such technical variations, including pipetting errors or slight inaccuracies in the initial DNA quantification, are common and can lead to a miscalculation of the slope, thereby yielding an efficiency value marginally above 110%. Despite this, the high coefficient of determination (R² > 0.99) of the standard curve confirms excellent linearity, and the distinct separation between positive and negative results validates the reliability of our data for qualitative prevalence assessment.

In conclusion, a highly sensitive, specific, and reproducible fluorescence qPCR method was successfully established for the rapid detection and quantification of *L. intracellularis*. This study provides the first systematic insight into the prevalence and distribution of *L. intracellularis* in Tibetan pigs from Xiangcheng County, offering critical scientific evidence and epidemiological data to support early monitoring, precise prevention and control, and health management of this pathogen in this unique local breed.

## Data Availability

The original contributions presented in the study are included in the article/supplementary material, further inquiries can be directed to the corresponding author/s.
